# Intraocular Epithelial Ingrowth after Traumatic and Surgical Corneal Injuries

**DOI:** 10.3390/diagnostics14131401

**Published:** 2024-07-01

**Authors:** Joana Heinzelmann, Sergiu Stoica, Alexander Ruben Vogt, Sabine Hecht, Anja Viestenz, Arne Viestenz

**Affiliations:** Department of Ophthalmology, UMH, Martin-Luther-University Halle-Wittenberg, 06120 Halle (Saale), Germany

**Keywords:** epithelial ingrowth, anterior optical coherence tomography (AS-OCT), cytokeratin 3, cytokeratin 13, cytokeratin 19, diagnosis

## Abstract

Intraocular epithelial ingrowth is a rare but serious complication of eye surgery or trauma. The diagnosis is challenging but can be aided by techniques such as anterior optical coherence tomography (AS-OCT). This study aimed to compare clinical and histopathological diagnostic data to evaluate the accuracy of AS-OCT. This retrospective analysis focused on patients presenting with suspected epithelial invasion between 2017 and 2022. Clinical data and histopathological findings were analyzed for diagnostic confirmation. Immunohistochemistry using the corneal-specific marker cytokeratin 3 and the conjunctival-specific marker cytokeratin 13 helped to identify the type of invading epithelial cells. Statistical analysis was used to evaluate the effectiveness of AS-OCT for diagnosis. This study included 51 patients who underwent keratoplasty or enucleation. Sixteen cases (31%) of epithelial ingrowth were histologically confirmed. The most common type was the cystic form (82%). Enucleation was required in 25% of patients, mainly due to diffuse epithelial invasion. Immunohistochemistry revealed a conjunctival origin in 82% of the infiltrated cells. AS-OCT showed a sensitivity of 78% and a specificity of 26% in detecting hyperreflective structures associated with epithelial invasion. This study highlights the diverse manifestations of epithelial ingrowth and the need for improved diagnostic techniques, with AS-OCT showing promising results but requiring further validation to avoid misdiagnosis.

## 1. Introduction

Intraocular epithelial ingrowth, also known as epithelial invasion and epithelial downgrowth, is a rare but serious complication following trauma [1,2,3] or eye surgery, including cataract extraction, penetrating or perforating keratoplasty, glaucoma surgery, vitrectomy and other procedures [3,4,5]. During this process, nonkeratinized epithelial cells invade through a traumatic or surgical wound and proliferate in the intraocular structures. Cataract surgery (either extracapsular or intracapsular) has been described as the most common cause of epithelial ingrowth, with a reported average incidence ranging from 0.076% to 0.12% [5,6]. The incidence of this condition after penetrating keratoplasty has been reported as 0.25% [7]. Most cases of epithelial ingrowth present within the first year following intraocular surgery, but there have been reports of cases presenting decades after surgery or trauma [8,9].

Epithelial cells from the cornea or conjunctiva penetrate the cornea or anterior chamber through a perforating injury and cause permanent damage to the eye, thereby impairing the ocular function. Based on the localization of the invading cells, a distinction is made between the intracorneal and retrocorneal locations. A difference must be made between the diffuse and cystic forms based on the type of growth. The diffuse form is described as more aggressive, as it can spread to intraocular structures and more frequently causes severe complications such as secondary glaucoma. In rare cases, epithelial invasion can occur as a mixture of both forms. Although the extensive spread of epithelial cells into the anterior chamber involving the adjacent iris, trabecular meshwork, ciliary body and retina is rare, it can lead to loss of vision [10].

A reliable diagnosis of epithelial invasion is difficult due to the presence of nonspecific signs and symptoms. Patients report decreasing visual acuity, photophobia, redness, tearing, and persistent pain in advanced stages [10,11]. Decompensation of the endothelium, the presence of a retrocorneal membrane, glaucoma, a positive Seidel test result and tractional retinal detachment are frequently observed clinically [10,11]. However, the extent of intraocular epithelial invasion can usually only be conclusively assessed by histopathological examination of surgical specimens using specific epithelial markers.

Several case reports have described the use of anterior optical coherence tomography (AS-OCT) as a noninvasive imaging technique to diagnose intraocular invasion by detecting hyperreflective structures [12,13]. 

Here, we present a monocentric, retrospective study of suspected cases of epithelial invasion with the aim of comparing clinical and histopathological data for the diagnosis and characterization of potential intraocular epithelial invasion and evaluating the accuracy of AS-OCT as a noninvasive imaging modality.

## 2. Materials and Methods

### 2.1. Cohort and Study Design

This study presents a retrospective monocentric analysis of all patients from the Department of Ophthalmology, University Hospital Halle (Saale), Germany, with suspected epithelial invasion and available clinical data and histopathological verification between 2017 and 2022. The patients’ data were analyzed anonymously. For diagnostic confirmation, representative formalin-fixed and paraffin-embedded (FFPE) tissue samples of the surgical specimens were histopathologically analyzed by the Institute of Pathology of University Hospital Halle (Saale), Germany, including hematoxylin and eosin (HE), periodic acid-Schiff (PAS), and keratin 19 (K19) staining. A Cirrus 6000 device (Carl Zeiss Meditec AG, Jena, Germany) was used for AS-OCT imaging. Only recent AS-OCT scans with a maximum period of 30 days before keratoplasty or enucleation were included in this study. Complete data sets comprising clinical data (including medical history and slit lamp photographs) and AS-OCT data were available for 32 patients and were analyzed in a blind fashion by two ophthalmology experts.

A regional ethics committee approved the study (MLU Halle–Wittenberg ethics committee vote: 2021-105).

### 2.2. Immunohistochemistry

Additional fluorescence staining of representative tissue samples with keratin 13 (K13) and keratin 3 (K3) served as a first step in identifying conjunctival and corneal epithelial cells. Paraffin sections from human sclerocorneal tissue were incubated for one hour and subsequently dehydrated with xylene and decreasing ethanol concentrations, followed by the addition of distilled water. Antigen retrieval was performed using 10 mM sodium citrate solution (pH 6) for 40 min in a water bath at 95 °C. To block nonspecific binding, tissue sections were incubated with antibody diluent solution (Agilent Dako, Santa Clara, CA, USA) at room temperature for 45 min. Next, the tissue sections were incubated with primary antibodies at 4 °C overnight. After being washed three times with PBS, the membranes were incubated with secondary antibodies for 1 h at room temperature in the dark. Finally, after washing with PBS, the tissue sections were mounted with ProLong Gold Antifade Mountant and stained with the DNA stain DAPI (Thermo Fisher Scientific, Waltham, MA, USA). The following primary antibodies were used: mouse anti-keratin 3 (ab68260, Abcam, Cambridge, UK) and rabbit anti-keratin 13 (ab92551, Abcam Limited, Cambridge, UK). The donkey anti-rabbit Alexa Fluor 568 (Abcam Limited, Cambridge, UK) and goat anti-mouse Alexa Fluor 488 (Cell Signaling Technology, Danvers, MA, USA) secondary antibodies were used. 

### 2.3. Statistical Analyses

Statistical analyses were performed using IBM SPSS Statistics (version 28, IBM Corp., Armonk, NY, USA). To evaluate the usefulness of AS-OCT as a noninvasive diagnostic tool for epithelial invasion, the sensitivity, specificity, negative predictive value (NPV), positive predictive value (PPV) and accuracy were calculated using standard methods.

## 3. Results

### 3.1. Clinical Characteristics and Etiology of the Study Cohort

Between 2017 and 2022, 51 patients with a suspected diagnosis of epithelial ingrowth were included in this five-year study. A total of 83 tissue samples were collected during keratoplasty or enucleation. In 16 patients (31%), epithelial ingrowth of the anterior chamber was histologically confirmed with the epithelial marker K19. The median age of those affected at the time of diagnosis was 57 years, and the majority of the patients were men (4.3:1).

It should be noted that the exact cause of the occurrence of epithelial ingrowths is difficult to determine due to the nonspecific clinical signs and the delayed diagnosis of this complication. We defined the first invasive event as a possible cause of epithelial ingrowth, although we are aware that other events could also be considered. Eight patients (50%) had experienced an initial ocular trauma. Eight patients (50%) underwent surgical procedures—four with penetrating keratoplasty, two with cataract extraction, one with laser-assisted in situ keratomileusis (LASIK) surgery and one with multiple untraceable penetrating operations. The ratio of males to females was 7:1 in the surgical group and 6:2 in the trauma group. The median age was 58 years in the surgical group and 54 years in the trauma group.

The calculated median time between the first event and diagnosis was one year (range from 0 to 39 years). However, the patients in the surgical group had a significantly longer time between the potential event and diagnosis (9 years) than did the patients in the traumatic epithelial invasion group (0 years). Four of 16 eyes (25%) had to be enucleated due to epithelial ingrowth. Notably, the epithelial ingrowth showed an absence of diffuse sheet layers in only one patient. In this case, enucleation was performed due to endophthalmitis. The clinical data of these patients are summarized in Table 1.

### 3.2. Immunohistochemical Characterization of Epithelial Ingrowth

**Figure 1:**
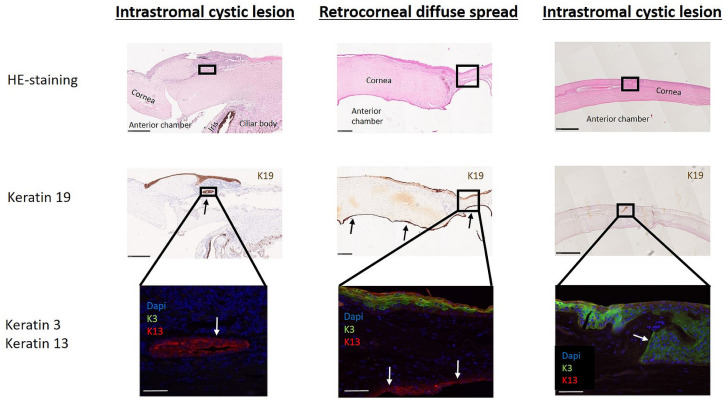
Microscopic images of immunohistopathological staining of human corneal tissues with verified epithelial ingrowth. Arrows indicate invaded epithelial cells. Scale bar: black = 500 µm, white = 20 µm.

Currently, histological examination using epithelial markers is the only diagnostic confirmation. Therefore, epithelial ingrowth was examined using antibodies against keratin 19 (K19), a protein that has been described and reported to be synthesized in epithelial cells of the conjunctiva and cornea [14]. A total of 83 postoperative specimens were available for histopathological examination, including 37 tissue samples from 16 patients with confirmed epithelial ingrowth. Histologic examination of the tissue samples revealed the following distribution: epithelial cells were detected intracorneally and retrocorneally in 11 patients (68.75%) and four patients (25%), respectively; for one of the patients (6.25%), the epithelial ingrowth evolved from intracorneal to retrocorneal during the treatment. Regarding the type of invasion, cystic epithelial ingrowth and diffuse epithelial ingrowth were diagnosed in 13 patients (81.25%) and two patients (12.5%), respectively. One patient (6.25%) had a mixed type, that is, primarily cystic invasion and, secondarily, diffuse epithelial invasion. Figure 1 shows an example of an intracorneal cystic lesion and retrocorneal diffuse epithelial ingrowth.

All the included samples were confirmed to be K19 positive by our Institute of Pathology, meeting the inclusion criteria for the study. To identify the cellular phenotype of the invading cells, further fluorescence staining was performed using K13 as a marker for conjunctival epithelial cells and K3 as a marker for corneal epithelial cells. Since, in most cases, only limited tissue material was available, the origin of the infiltrated epithelium could only be analyzed in 13 tissue samples from 11 patients. It should be noted that the superficial epithelial layer of the normal human cornea was positive for both cytokeratins (K13 and K3), but the human conjunctival epithelium was stained only with an anti-K13 antibody. Nine patients (82%) were strongly positive for K13 alone, as exemplarily demonstrated in Figure 1, suggesting epithelial ingrowth of conjunctival origin. Analysis for K3-positive cells revealed that two patients had epithelial invasion of corneal origin. One patient (9%) was strongly positive for both markers, K3 and K13, and one patient (9%) was positive for K3 alone (Table 2).

### 3.3. Evaluation of Optical Coherence Tomography of the Anterior Segment of the Eye (AS-OCT) for the Diagnosis of Epithelial Ingrowth

To prove the validity of AS-OCT as an alternative noninvasive imaging tool for diagnosis, we evaluated AS-OCT scans of patients with potential epithelial ingrowth. Therefore, suitable AS-OCT images were included in this study for 32 of 83 specimens with a suspected diagnosis of epithelial ingrowth. Two experienced ophthalmologists evaluated the AS-OCT images in a blind manner to detect the possible presence of intraocular epithelial cells. Further details of the patients’ medical histories and available information from further noninvasive diagnostic tools from routine ophthalmologic practice were included in this assessment. In the AS-OCT scans, the intracorneal or retrocorneal presence of hyperreflective structures was determined.

**Figure 2:**
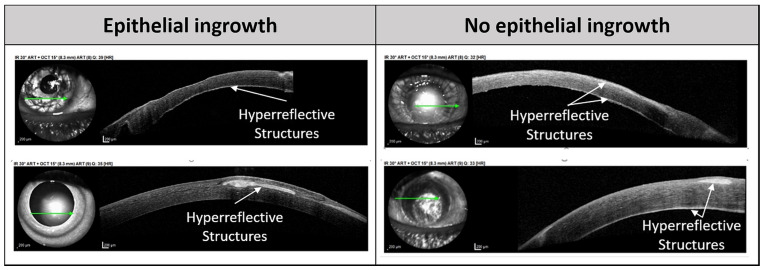
AS-OCT images of human corneas with and without histological confirmed epithelial ingrowth. Arrows indicate hyperreflective structures.

AS-OCT analysis revealed suspected hyperreflective structures in 24 of the 32 patients (Figure 2). The correlation with histologically confirmed epithelial invasion showed a sensitivity of 78% and a specificity of 26%. The PPV was 29%, and the NPV was 75%. Hyperreflective structures were differentially diagnosed as keratitis and other keratopathies in 45%, corneal scars and pannus in 25%, corneal transplant rejections in 20% and corneal ulcers in 10% of the patients.

## 4. Discussion

Intraocular epithelial invasion is an uncommon but difficult disease to diagnose due to its nonspecific signs and symptoms and delayed epithelial ingrowth. Untreated or late-diagnosed epithelial ingrowth can lead to blindness and/or loss of the affected eye. Nonspecific signs include a retrocorneal membrane, increased intraocular pressure, leakage or corneal edema. Additionally, the medical history may also be indicative. Thus, the ingrowth of corneal or conjunctival epithelial cells can be caused by incomplete or delayed wound healing after penetrating eye injuries or intraocular surgeries, incarceration of the iris into the wound, or the transfer of viable epithelial cells at the time of surgery [15]. Based on this knowledge, we identified 51 suspected cases of epithelial invasion of the anterior chamber in our retrospective analysis between 2017 and 2022 in our hospital. In 16 patients (31%), the epithelial invasion was confirmed histologically. Among these patients, eight experienced an initial ocular trauma, with three cases caused by direct contact with chemicals that induced chemical burns on the cornea, two by ulcers, two by direct penetration with metal objects, and one by penetration with a wooden stick. All of the traumas exhibited a high degree of severity, necessitating immediate medical treatment. However, due to the primary care being administered either at different clinics or many years before the diagnosis of epithelial ingrowth, a clear correlation between the severity of the injury and the extent of epithelial ingrowth or the probability of enucleation could not be established. Regarding the surgical causes, four cases occurred after penetrating keratoplasty, two cases after cataract extraction, one case after LASIK surgery and one patient had multiple prior surgeries that were untraceable and performed at external facilities. In former publications, epithelial invasion has been described to occur mainly after cataract surgery and ocular trauma, but keratoplasty and other procedures, including LASIK, may also be the cause [11,12,13,16]. Although this was a small case series, we suspect that the low number of cataract operations involved in our study is due to the significant improvement in surgical methods in recent years, including advanced surgical techniques and equipment.

Limited immunohistochemical analyses have allowed the identification of the original localization of invading epithelial cells. Corneal and conjunctival cells are characterized by the expression of different cytokeratins, which are keratin antigens of intermediate filaments. Cytokeratins can be divided into two subfamilies: acid type including K9-20 and neutral to basic type including K1-8. Their function is not only to maintain the shape of a cell and protect the cell from mechanical stress but also differentiation and specialized functions. The tissue-specific and differentiation-specific expression of the various cytokeratins can help to determine the presence of human corneal and conjunctival epithelial cells. In this study, we used K19 as a marker to confirm the diagnosis of epithelial ingrowth, which is routinely used for this purpose in our hospital. On the human ocular surface, K19 is described to be expressed in the peripheral, corneal, basal epithelial cells of the limbus and in the conjunctiva [17,18,19]. We performed immunohistochemistry against K3 and K13 in addition to hematoxylin and eosin staining to differentiate the corneal and conjunctival epithelia. K3 specifically stains only the human corneal epithelium but not the conjunctiva [20,21]. In contrast to those of the cornea, epithelial cells of the human conjunctiva are reported to be specifically positive for K13 and negative for K3 [17]. However, it should be noted that CK13 is also expressed in the peripheral corneal epithelium [19]. Based on this knowledge, we used these epithelial markers to provide initial insight into the phenotype of the invading cells. Our immunohistochemistry analyses of excised tissue showed that the intrastromal or retrocorneal epithelial cells were positive for K13 and negative for K3 in most cases. These findings indicate that the source of the epithelium for ocular ingrowth was mainly conjunctival, which is consistent with previous observations [11,22,23,24]. 

Epithelial ingrowth can occur in the form of cysts or diffuse sheet-like layers. In six surgical patients and six traumatic patients, the epithelial ingrowth was cystic. Diffuse epithelial ingrowth was detected in one of the surgical and traumatic cases. One patient with a history of trauma developed a combination of both diffuse and cystic forms. In our cohort, the enucleation rate was 25%. Two of the four enucleated bulbs exhibited sheet-like diffuse epithelial ingrowth, and one patient developed an initial cystic form followed by the diffuse form. In one of the enucleated eyes, initial cystic epithelial ingrowth was confirmed, but it should be noted that the reason for enucleation was endophthalmitis. Thus, our data confirm the findings of previous reports that diffuse epithelial ingrowth tends to be more aggressive, is difficult to detect and has a greater risk of recurrence after treatment [11,25]. Epithelial cysts usually proliferate slowly with a defined spread and can be detected easily on clinical examination, which allows surgical removal of the epithelium, with a more benign clinical course and a better prognosis. To prevent ingrowth of the epithelium, prompt and careful closure of the wound edges and meticulous care of the incisions during and after the surgical procedure are crucial. Wound leaks should be evaluated and repaired if necessary.

The diagnosis of epithelial ingrowth can be challenging because of its unspecific symptoms and signs. Currently, histological analysis of surgically removed tissue is the most accurate method for diagnosing this disease. Noninvasive diagnostic techniques can help to evaluate suspected epithelial invasion in an early stage and monitor the clinical efficacy of treatment. Previously described noninvasive test methods include slit lamp examination, specular microscopy, diagnostic argon laser photocoagulation and confocal microscopy. However, specular microscopy is limited by the presence of corneal edema [26], and argon laser photocoagulation is only useful if the epithelium is present on the iris surface [27] but may lead to opening of the cyst and the spreading of epithelial cells. In vivo confocal laser microscopy is another promising, sensitive imaging tool for detecting epithelial ingrowth [12,28,29] but has drawbacks due to its limited penetration depth; it is a contact imaging modality that can lead to further complications, and expert knowledge is required for data interpretation. AS-OCT has been described as yet another promising noninvasive and noncontact imaging tool [12,13]. This imaging technique can be used to visualize the different layers of the cornea, sclera, chamber angle and iris in the anterior chamber in cross-sections. Suh et al. showed that intracorneal, retrocorneal or intracameral white hyperreflective layers can indicate the presence of invading epithelial cells [13]. To our knowledge, our study represents the largest cohort to evaluate AS-OCT as a tool for the diagnosis of epithelial ingrowth. Based on 32 cases of suspected epithelial ingrowth, we showed that the blind evaluation of the AS-OCT scans by experienced ophthalmologists with the aid of the available clinical data of the patients had a relatively high sensitivity and NPV. Thus, we confirmed further observations, also describing hyperreflective areas to be associated with this posttraumatic or postoperative event [12,30,31,32]. However, the specificity and PPV were relatively low, implying a high risk of misdiagnosis of epithelial invasion when used exclusively. Therefore, it is important to note that AS-OCT can provide important initial indications of potential epithelial invasion but cannot be used as conclusive proof. It must be considered in the differential diagnoses that hyperreflective structures can also occur in other pathologies, such as keratitis and other keratopathies, as well as corneal scars, corneal transplant rejection, corneal dystrophies and corneal ulcers [33,34]. Thus, AS-OCT provides comprehensive and reproducible information on the anterior chamber, helping clinicians identify disorders of the cornea and ocular surface, including epithelial ingrowth. However, further diagnostic tools are necessary to ensure an accurate diagnosis.

## 5. Conclusions

In summary, our retrospective study identified a case series of epithelial ingrowths originating mainly from conjunctival cells. Epithelial ingrowth can manifest as cysts or diffuse layers, with the latter being more aggressive. AS-OCT shows promise for diagnosis of this disease, but further validation is needed due to limitations in its specificity and positive predictive value. Overall, although AS-OCT provides valuable initial findings, further diagnostic tools are essential for confirmation.

## Figures and Tables

**Table 1:** Characteristics of patients with epithelial ingrowth.

Patients with Epithelial Ingrowth, *n*	16
Trauma/surgery, *n* (%)	8/8 (50/50)
Median age of the patients, in years (min, max)	57 (2–94)
Surgical patients	58 (39–73)
Trauma patients	54 (2–94)
Male/female, *n* (%)	13/3 (81/19)
Surgical patients	7/1 (87/13)
Trauma patients	6/2 (75/25)
Median time between event and diagnosis, in years (min, max) ^1^	1 (0–39)
Surgical patients	9 (0–39)
Trauma patients	0 (0–16)
Enucleated eyes, *n* (%)	4 (25)
Diffuse/cystic	3/1
Retrocorneal/intrastromal	3/1

^1^ Time was calculated from the first invasive event until the diagnosis.

**Table 2:** Immunohistological staining of K19+ tissue samples.

Patients with Epithelial Ingrowth, *n*	11
K3−/K13+, *n* (%)	9 (82%)
K3+/K13−, *n* (%)	1 (9%)
K3+/K13+, *n* (%)	1 (9%)

## Data Availability

The datasets generated during and/or analyzed during the current study are available upon request from the corresponding author.
